# Electronic Clinical Decision Support Tools: Strategies to Improve the Management of Lower Respiratory Tract Infections in Low-Resource Settings

**DOI:** 10.4269/ajtmh.24-0126

**Published:** 2024-10-08

**Authors:** L. Gayani Tillekeratne, Warsha De Soyza, Maria D. Iglesias-Ussel, Stefany Olague, Dhammika Palangasinghe, Ajith Nagahawatte, Thilini Wickramatunga, Jayani Gamage, Ruvini Kurukulasooriya, Madureka Premamali, James Ngocho, Armstrong Obale, Kate Sanborn, John Gallis, Christopher W. Woods, Susanna Naggie, Truls Ostbye, Hrishikesh Chakraborty, Eric Laber, Evan Myers, Melissa Watt, Champica K. Bodinayake

**Affiliations:** ^1^ Duke University School of Medicine, Department of Medicine, Durham, North Carolina;; ^2^ Duke Global Health Institute, Durham, North Carolina;; ^3^ Faculty of Medicine, Department of Medicine, University of Ruhuna, Galle, Sri Lanka;; ^4^ Ruhuna-Duke Centre for Infectious Diseases, Faculty of Medicine, University of Ruhuna, Galle, Sri Lanka;; ^5^ Duke Clinical Research Institute, Durham, North Carolina;; ^6^ Faculty of Medicine, Department of Microbiology, University of Ruhuna, Galle, Sri Lanka;; ^7^ Kilimanjaro Christian Medical Centre, Moshi, Tanzania;; ^8^ Duke University School of Medicine, Department of Family Medicine and Community Health, Durham, North Carolina;; ^9^ Duke University School of Medicine, Department of Biostatistics and Bioinformatics, Durham, North Carolina;; ^10^ Duke University School of Medicine, Department of Obstetrics & Gynecology, Durham, North Carolina;; ^11^ University of Utah, Salt Lake City, Utah

## Abstract

Lower respiratory tract infection (LRTI) is a common reason for hospitalization and antibacterial use globally. There is considerable overlap in the clinical presentation of bacterial and viral LRTIs. Low- or middle-income countries (LMICs) face the dual challenge of appropriately targeting antibacterials for bacterial LRTI while reducing inappropriate antibacterials for viral LRTI. We propose a framework by which an electronic clinical decision support tool (eCDST) for diagnosing LRTI and reducing unnecessary antibacterial use may be developed, validated, and prospectively evaluated in an LMIC. The developed tool would be data driven, low-cost, feasible in the local setting, adaptable based on resource availability, and updated in real time, with prospective assessment to identify its clinical impact. We draw upon our team’s recent experience developing an eCDST for LRTI management in Sri Lanka. Publicly sharing such processes and data is valuable, such that we can collectively improve clinical care in LMICs and other settings.

## INTRODUCTION

Lower respiratory tract infection (LRTI), including syndromes such as bronchitis, infective exacerbations of chronic obstructive pulmonary disease, and pneumonia, is one of the most common reasons for hospitalization globally.^1^ Historically, bacterial etiologies of LRTI have been the most feared owing to the possibility of fatal outcomes if misdiagnosed and undertreated.^2^ The bacterium *Streptococcus pneumoniae* remains the most common cause of LRTI and LRTI mortality.^3^ However, the advent of molecular diagnostics has shown that viruses are an important cause of LRTI and are often more commonly identified than bacteria as the primary etiology of infection in both children and adults.^4^^–^^7^ The true etiology of LRTI typically remains unknown in over 50% of patients in both clinical practice and research studies, given limitations associated with current diagnostics and factors such as prior antibiotic use.^1^^,^^2^^,^^5^

Despite the high prevalence of viral LRTIs, antibacterial overuse for LRTI remains high, especially in a low- or middle-income country (LMIC) settings. The recent pandemic of coronavirus disease 2019 has exacerbated the overuse of antibacterials for viral LRTIs.^8^^–^^10^ The unnecessary use of antibacterials results in negative patient outcomes and fuels downstream antibacterial resistance, which was associated with over 4 million deaths globally in 2019.^11^^,^^12^ Most of the deaths due to antibiotic-resistant infections are in LMICs, including sub-Saharan Africa and South Asia, which also bear the greatest burden of LRTIs.^11^^,^^13^ The LMICs thus face the dual challenge of appropriately targeting antibacterials for bacterial LRTIs while reducing inappropriate antibacterials for viral LRTIs.

### Results from our setting regarding LRTI treatment.

Since 2012, our team has been investigating the epidemiology, diagnosis, and management of acute respiratory tract infections in southern Sri Lanka.^14^^–^^21^ We have shown that LRTI is the most common indication for antibacterial use in the inpatient setting for both children and adults.^22^ Similarly, we have shown that respiratory tract infections are the most common indication for antibacterial use among outpatients.^23^ From 2018 through 2021, we conducted the Respiratory Infection Severity and Etiology in Sri Lanka study, enrolling over 1,000 hospitalized children and adults meeting a clinical case definition for LRTI. We conducted comprehensive etiological testing, including blood cultures, sputum cultures, multiplex polymerase chain reaction (PCR) testing of nasopharyngeal samples for respiratory pathogens, and urine antigen testing for *S. pneumoniae*, as well as multiplex PCR testing of sputum samples for respiratory pathogens among a subset. Physicians with experience in clinical medicine and infectious diseases reviewed clinical and laboratory data, imaging findings, and etiological testing results to clinically adjudicate the etiology of illness for each patient. Approximately half of our cohort did not have a clear etiology of illness identified, as is commonly seen with LRTIs.^1^^,^^2^^,^^5^ However, among those who did have an etiology identified, approximately 50% had a viral etiology, 25% had a bacterial etiology, and 25% had a noninfectious etiology. Over 80% of children and adults with viral etiologies and noninfectious etiologies (as well as bacterial etiologies) received treatment with antibacterials, suggesting a high prevalence of inappropriate antibacterial use in our setting.^24^ Low-cost interventions to better diagnose LRTI etiology and target antibacterial treatment may be helpful in our setting and other similar LMIC contexts.

### Algorithm-based tools to improve antibacterial prescribing for clinical syndromes.

Algorithm-based tools to improve antibacterial prescribing have been developed for LMICs, especially for acute febrile illness (AFI). These tools are largely based on using patient-level data, with newer strategies incorporating point-of-care (POC) testing to alter the probability of a certain pathogen causing disease. The Integrated Management of Childhood Illness (IMCI) and Integrated Management of Adolescent and Adult Illness (IMAI) approaches by the WHO were among the first algorithm-based tools developed for LMICs and were created to provide basic syndromic definitions for diagnosis and treatment.^25^^–^^27^ However, the IMCI/IMAI do not capture local epidemiology well, if at all, and are designed to maximize sensitivity for bacterial infection over specificity, with the result that patients may be overtreated with antibacterials.^28^^,^^29^

With advancements in science and enhanced capacity in LMICs, newer strategies that incorporate low-cost POC diagnostics into algorithms have recently been developed. A multifaceted intervention consisting of POC tests (for influenza A and B, respiratory syncytial virus, group A *Streptococcus*, *S. pneumoniae*, dengue, typhoid, malaria, white blood cell count with differential, and C-reactive protein [CRP], as well as a urinalysis), a diagnostic algorithm, and a training and communication module were recently assessed in several interventional clinical trials among patients with AFI in sub-Saharan Africa.^30^ The trials showed mixed results of the intervention on decreasing antibacterial use. Among children 6 months–18 years of age in Burkina Faso, the intervention resulted in a decrease in antibacterial use compared with the control group (40.6% versus 57.5%; *P* <0.001).^31^ Similarly, among children 6 months–18 years of age in Ghana, the intervention resulted in a statistically significant relative risk reduction of 11% in antibiotic prescriptions.^32^ However, among patients ≥1 year of age in Uganda, there was no difference in antibacterial prescribing in the intervention group compared with the control group.^33^ A meta-analyses of these results suggested that a reduction in antibacterial prescriptions was especially pronounced in those with respiratory symptoms, without malaria, and who were <5 years of age.^34^ White blood cell count and differential, CRP, and malaria testing were applied systematically across intervention groups in all three countries, whereas the seven other POC tests were applied variably based on patients’ symptoms and test availability. These results suggest that locally relevant algorithms, or at least universal algorithms that are adapted to local conditions, as well as an understanding of how POC tests are used are important in developing effective algorithms for an LMIC setting.

### Electronic clinical decision support tools to improve antibacterial prescribing.

The implementation of algorithms in easy-to-use electronic formats has the potential to help overcome low compliance with traditional paper-based algorithms.^35^ The increased used of data and artificial intelligence, as well as the ubiquity of mobile phones and cellular connections, has fueled the rise of electronic clinical decision support tools (eCDSTs), which can support healthcare professionals in their daily clinical decision-making.^36^ Electronic tools may be especially useful in LMICs given low physician-to-patient ratios and limited laboratory infrastructure for complex testing.^36^

A few studies have assessed the impact of eCDST-based algorithms on antibacterial prescription. In Afghanistan and Nigeria, observational studies demonstrated that the use of ALMANACH (Algorithms for the Management of Acute Childhood Illness), an electronic version of the IMCI, could result in a decrease in antibacterial prescriptions in children.^37^^–^^39^ A few studies in LMICs have implemented an eCDST and prospectively assessed its impact on clinical care and antibacterial use in randomized clinical trials and have also shown mixed results. In Tanzania, Keitel et al.^40^ developed a novel electronic algorithm named e-POCT, which consists of clinical signs and POC tests (hemoglobin, glucose, oximetry, malaria, CRP, and procalcitonin). In children 2–59 months of age presenting with an AFI, they found that e-POCT was safe and reduced antibacterial use to 11.5% compared with 29.7% in the control group using ALMANACH.^40^ The team then developed the ePOCT+, which expanded the target population to children <15 years of age presenting with any acute medical or surgical condition and uses a combination of clinical signs and POC tests (hemoglobin, oximetry, and CRP). In a cluster-randomized trial in Tanzanian primary care facilities, the use of the ePOCT+ in conjunction with mentorship support resulted in a reduction in antibiotic prescriptions to 23.2% compared with 70.1%, with noninferior clinical outcomes at day 7.^41^ Conversely, in Burkina Faso, the use of an electronic integrated eDiagnosis approach developed in line with ICMI guidelines did not improve overall correct medication prescriptions based on healthcare workers’ diagnoses but may have reduced antibiotic overprescription.^42^ The use of eCDSTs designed specifically to improve antibacterial prescription for LRTIs has not been explored in LMICs.

### Proposed framework for developing an eCDST for managing LRTIs in an LMIC setting.

Our team has embarked on an effort to develop and then evaluate an eCDST for LRTI management in Sri Lanka that integrates population-level, real-time surveillance data, patient-level clinical predictors, POC pathogen tests, and POC biomarker tests. By integrating real-time surveillance data for viral pathogens that are circulating locally, we aim to develop a model that is responsive to changing epidemiology and potential outbreaks. Patient-level clinical predictors will be derived from the biorepository of patients with LRTIs that we previously enrolled in Sri Lanka from 2018 to 2021 and may include demographic characteristics, exposures, clinical symptoms, physical exam findings, and results from basic laboratory tests such as complete blood counts. Point-of-care pathogen and biomarker tests will also be integrated into the algorithm, with inclusion of locally approved tests whenever possible. Machine learning techniques will be applied to select the key variables from these components to develop a diagnostic and treatment algorithm for LRTIs. The results will be output as decision lists, which are represented as a sequence of human-readable if-then statements.^43^^–^^45^ We will optimize this algorithm by incorporating costs, such that the list that maximizes diagnostic accuracy while minimizing cost is identified. The algorithm will then be internally validated within the data and translated to an electronic application that is easily accessible and usable on physicians’ mobile phones. We will rely on iterative cycles of stakeholder input to refine the tool. Prior to developing our application, we will conduct interviews with Sri Lankan physicians to identify barriers and facilitators to using an LRTI diagnostic and treatment tool, to better understand their needs and preferences. We will then develop an initial version of our eCDST and conduct several rounds of focus group discussions with the same physicians to iteratively improve our eCDST based on their feedback. We will model our qualitative work on two behavioral theory frameworks, the Capability, Opportunity, Motivation, and Behavior model and the Theoretical Domains Framework, to allow us to identify influences on behavior that affect implementation of strategies.^46^^,^^47^ Finally, we will evaluate the impact of the eCDST on antibacterial use and clinical outcomes by prospectively conducting a randomized controlled trial in Sri Lanka.

The digital health landscape in Sri Lanka is still nascent and generally limited to localized and siloed medical record systems. However, the Sri Lanka Ministry of Health developed its first Digital Health Blueprint in 2023, with the plan to eventually digitalize clinical workflows, share information between digital health systems, and connect health systems to the broader digital ecosystem. The Ministry’s ultimate goal is to use evidence-based processes and real data to develop innovative clinical processes and transform the health system of Sri Lanka into a more efficient, patient-centric, high-quality system.^48^ We hope that the proposed eCDST is a prototype that can be augmented in the future for using electronic health data generated in real time in the local setting.

### Characteristics that are important for eCDSTs to improve antibacterial prescription in LMIC settings.

There are multiple principles that our team, consisting of researchers from Sri Lanka and the United States, have strived to follow when developing an eCDST for LRTI management in an LMIC. Many of these principles have been described previously and are in line with the Principles for Digital Development, a set of guiding tenets that were devised to promote sustainable and inclusive development in the global digital landscape and have been endorsed by over 300 organizations, including the WHO.^49^ First, the evidence base on which the eCDST is derived must be locally generated and relevant. Many eCDSTs are developed based on data from patients living in different climates and environments or higher-resourced settings; however, applying these rules directly to patients in LMIC settings may not be appropriate.^36^ For tool content and development, the evidence should be high quality and should originate from similar cohorts, similar environments, or well-established data sources.^50^ Second, validation of content and structure is important and should be well documented such that output is accurate and reproducible and that patient safety can be ensured.^35^^,^^50^ Third, an algorithm that recommends treatment decisions should be easy to use and interpret, meaning that the clinician can understand how input data are processed into output data.^50^ Furthermore, physicians should have the ability to bypass diagnostic tests based on resource availability, and therapeutic recommendations should be based on locally available drugs and local or national treatment guidelines.^50^

There is little regulatory guidance in LMICs about eCDST implementation, but we have strived again to follow previously described principles when preparing for implementation.^35^ For one, diagnostic tools incorporated into eCDSTs should be compliant with local regulations.^50^ In addition, eCDSTs should be accessible through apps downloaded on compatible devices, which can be feasibly accessed by clinicians.^50^ Features such as the ability to use an eCDST offline and sync data when connectivity is available, protection against cyberattacks, and compliance with local data policies and legislation are also important.^50^ Allocating resources efficiently is especially important in LMIC settings, and an assessment of cost and cost-effectiveness should be factored into eCDST development and implementation. Most eCDSTs have been evaluated retrospectively, and few have been evaluated in randomized controlled trials, which are considered the gold standard for evaluating impact.^36^ Effectiveness in the local, real-world setting should be evaluated, and once implemented at scale, health system impacts such as supply chain and costs should be explored.^35^ Finally, throughout the development and implementation process, it should be emphasized that eCDSTs are only meant to complement the clinician’s role. Clinical judgment and experience, which may take into account factors such as comorbidities pertinent to a specific patient, should remain paramount. Such messaging may ameliorate any worries that may decrease uptake of eCDSTs and adherence to provided guidance.

## CONCLUSION

Lower respiratory tract infection and antimicrobial resistance pose dual challenges in LMIC settings. The potential exists to leverage recent developments in affordable mobile technology and computational techniques, such as machine learning, to positively impact both of these conditions. We propose a framework by which an eCDST for diagnosing LRTIs and reducing unnecessary antibacterial use can be developed, validated, and prospectively evaluated in an LMIC. The developed tool would be data driven, low-cost, feasible in the local setting, adaptable based on resource availability, and updated in real time, with prospective assessment to identify the clinical and public health impacts of the tool. Public sharing of such processes and data is valuable for iterating on tools, such that we can collectively improve clinical care in LMIC and other settings.
